# Time trends in cardiovascular disease mortality attributable to non-optimal temperatures in China: An age-period-cohort analysis using the Global Burden of Disease Study 2019

**DOI:** 10.3389/fpubh.2022.1075551

**Published:** 2023-04-05

**Authors:** Jiehua Wei, Peiwen Wang, Fan Xia, Junxiang Miao, Xuan Zhou, Ziqi Yang, Ziqiang Gong, Lizhang Chen, Tingting Wang

**Affiliations:** Department of Epidemiology and Health Statistics, Xiangya School of Public Health, Central South University, Changsha, Hunan, China

**Keywords:** cardiovascular diseases, myocardial ischemia, stroke, mortality, non-optimal temperatures, age-period-cohort model

## Abstract

**Background:**

Associations between non-optimal temperatures and cardiovascular disease (CVD) mortality risk have been previously reported, yet the trends of CVD mortality attributable to non-optimal temperatures remain unclear in China. We analyzed trends in CVD mortality attributable to non-optimal temperatures and associations with age, period, and birth cohort.

**Methods:**

Data were obtained from the Global Burden of Disease Study (GBD) 2019. Joinpoint regression analysis was used to calculate annual percent change (APC) and average annual percent change (AAPC) from 1990 to 2019. We used the age-period-cohort model to analyze age, period, and cohort effects in CVD mortality attributable to non-optimal temperatures between 1990 and 2019.

**Results:**

The age-standardized mortality rate (ASMR) of CVD attributable to non-optimal temperature generally declined in China from 1990 to 2019, whereas ischemic heart disease (IHD) increased slightly. Low temperatures have a greater death burden than high temperatures, but the death burden from high temperatures showed steady increases. Joinpoint regression analysis showed that CVD mortality decreased in all age groups except for IHD, and the decreases were greater in females than in males. The mortality of CVD attributable to non-optimal temperatures of males was higher than females. The mortality rate showed an upwards trend with age across all CVD categories. Period risks were generally found in unfavorable trends. The cohort effects showed a progressive downward trend during the entire period.

**Conclusion:**

Although there have been reductions in CVD mortality attributable to non-optimum temperatures, the mortality of IHD has increased and the burden from non-optimal temperatures remains high in China. In the context of global climate change, our results call for more attention and strategies to address climate change that protect human health from non-optimal temperatures.

## 1. Introduction

Cardiovascular disease (CVD), principally ischemic heart disease (IHD) and stroke, are the leading causes of death and disability-adjusted life years (DALYs) globally in 2019, especially in low-income and middle-income countries (1–3). As the Global Burden of Disease 2019 Study reported, there were ~523 million cases and 18.6 million deaths worldwide caused by CVD in 2019 (2, 4). With the largest population in the world, China had always experienced a heavy health and economic burden of CVD due to population aging and the increasing prevalence of many risk factors (5, 6). In 1990, stroke and IHD were ranked as the 3rd and 7th leading causes of DALYs in China; in 2017, they rose higher to the 1st and 2nd leading cause, respectively (5). CVD is caused by various risk factors, mainly including metabolic factors (i.e., lipids, diabetes, obesity, and hypertension), behavioral factors (i.e., tobacco use, alcohol, diet quality, and physical activity), socioeconomic and psychosocial factors (i.e., education, depression), and environmental factors (i.e., air pollution, ambient temperatures) (7–9).

Climate change will become the biggest health threat of the 21st century and an urgent problem to be solved, the Lancet Countdown to climate change has warned (10). In recent years, there are more and more extreme heat and cold waves around the world due to climate change, and it affects human health directly by increasing exposure to extreme temperatures. According to the GBD 2019 study, non-optimal temperatures are among the top 10 causes of death globally (11). There were 1.69 million deaths would attributable to non-optimal temperature globally in 2019, and low temperature has a greater overall effect on mortality than does high temperature (12). The epidemiological evidence suggests a U- or J-shaped association between ambient temperatures and the risk of death from cardiovascular and cerebrovascular, which means both low and high temperatures may increase the risk of mortality (13–15). Recently, a study reported that 399.7 thousand deaths who were diagnosed with CVD were attributed to non-optimal temperatures across China in 2019, indicating a substantial burden of CVD due to non-optimum temperatures (16). However, there are only 3 years left to achieve the target of reducing CVD mortality in China by 15% by 2025 compared with 2015, which was adopted in the document Medium- to Long-Term Plan for the Prevention and Treatment of Chronic Diseases (2017–2025) (17). In this context, understanding the CVD death and trends attributable to non-optimum temperatures in China is key to guiding CVD prevention and control efforts under climate change.

To date, previous analyses have focused on the association between CVD and non-optimal temperatures in China (18–20). None of the existing studies explore the long-term trends of CVD mortality attributable to non-optimal temperatures between different age groups and gender, and there is a lack of comprehensive analyses of the possible causes underlying the long-term trends. Therefore, in this study, we examine the effects of age, period, and cohort on CVD mortality attributable to non-optimal temperatures and the temporal trends from 1990 to 2019 in China, using data from the GBD 2019.

## 2. Materials and methods

### 2.1. Data source

The attributable burden of CVD data was obtained from the GBD 2019 study, which was provided by the Institute for Health Metrics and Evaluation (IHME). The GBD 2019 study used DisMod-MR 2.1, a Bayesian meta-regression tool as the primary method to comprehensively estimate disease burden (e.g., incidence, prevalence, mortality, and DALYs) for 369 diseases and injuries and 87 risk factors in 204 countries and territories from 1990 to 2019 (2, 11). Details of the data, methodology to enhance data quality and comparability, and statistical modeling for the GBD 2019 have been explained previously. All anonymized data have been publicly available on the website of IHME and can be accessed online (http://ghdx.healthdata.org/gbd-results-tool). The University of Washington Institutional Review Board reviewed and approved the informed consent. Original data of CVD mortality in China were mainly from Disease Surveillance Points, Maternal and Child Surveillance System, Chinese Center for Disease Control and Prevention Cause of Death Reporting System (21). IHD and stroke cases were classified using the International Classification of Diseases and Injuries, 10th Revision.

In the GBD 2019 study, the daily averages of temperature for each location were obtained from the European Center for Medium-Range Weather Forecasts. The theoretical minimum risk exposure level for temperature (TMREL), which meant the temperature associated with the lowest mortality risk for all included causes combined, was estimated for a given location and year. Given varying TMREL for different regions (e.g., higher in warm regions than colder locations), years, and diseases, the GBD study 2019 employed both spatially and temporally varying to estimate TMREL and are not using a globally uniform TMREL (11, 13, 22). Exposure to non-optimal temperature is defined as the same-day exposure to ambient temperature that is either warmer or colder than the temperature associated with the minimum mortality risk (11). High-temperature exposure is defined as exposure to temperatures warmer than this TMREL and low-temperature is defined as temperatures colder than this TMREL. The population attributable fraction (PAF) is defined as the meaning that, if the exposure to a risk factor is reduced to the theoretical minimum exposure level, then the proportion of associated disease or death in the population would decrease (i.e., the proportion of cause-specific deaths attributable to high or low daily temperatures). The PAF associated with non-optimal temperature is an aggregate of high-temperature and low-temperature PAFs in each location and year. We computed PAF by age-sex-location-year using the following general formula for a continuous risk:


(1) $$\begin{array}{l} PAF=\frac{\Sigma_{i}^{n}P_{i}\left(RR_{i}-1\right)}{\Sigma_{i}^{n}P_{i}\left(RR_{i}-1\right)+1} \end{array}$$

where *P*_*i*_ is the percentage of the population exposed to level *i* of high or low temperature, *n* is the total number of exposure level. *RR*_*i*_ is the relative risk as a function of exposure level *i* of high or low temperature, and was estimated based on 81 published systematic reviews, whose specific methods have been outlined previously (11).

The number of attributable deaths (ADs) was calculated by multiplying the PAFs with the number of CVD death cases (N) (23). It can be expressed as follows:


(2) $$\begin{array}{l} AD=PAF*N \end{array}$$

Age-standardized mortality rate (ASMR) and the 95% uncertainty intervals (UIs) was calculated using the GBD 2019 global standard population. The detailed methods were introduced in GBD 2019 report and the official website.

### 2.2. Statistical analysis

The rate of mortality with 95% UI of the CVD is reported according to age and gender. All the rates are reported per 100,000 population. Identifying changes in the secular trend is critical to analyzing disease mortality data. Joinpoint regression analysis was used to determine temporal trend changes of CVD mortality attributable to non-optimal temperature from 1990 to 2019. Annual percent change (APC) and 95% confidence interval (CI) were calculated for each mortality trend, and Average annual percent change (AAPC) and 95% CI were calculated for the full range of period analyzed. The APC and AAPC were used to describe the temporal trends of CVD mortality. Significant changes of the time points were tested using a Monte Carlo substitution method. The hypothesis test was whether AAPC/APC was significantly different compared to zero. An increasing trend was defined as APC/AAPC > 0, and a decreasing trend was defined as APC/AAPC < 0, vice versa. The analysis was carried out by the Joinpoint Regression Program software (version 4.9.0.1; Statistical Research and Applications Branch, National Cancer Institute).

The age-period-cohort model is a common statistical model to extract information hidden in mortality, including the risk of death experienced by the population in a given year and the accumulation of health risks since birth. This model allows the analysis of the independent effects of age, period, and cohort on temporal trends in the mortality of CVD. It has been used in the descriptive epidemiology of certain chronic diseases, including cardiovascular disease (24). The age effect represents the different risks in various age groups (25). Period effect reflects changes over time affecting non-optimal temperature-attributable CVD mortality in all age groups, presumably arising from changes in social, cultural, economic, or physical environments. Birth cohort effects reflect the characteristics of individuals with the same birth year and consider the risk factors and exposure to environmental factors present in early life. For age-period-cohort analyses, we arranged the mortality and population data into successive 5-year age groups from 25–29 years to 80–84 years, consecutive 5-year periods from 1990 to 2019, and correspondingly consecutive 5-year birth cohort groups starting from 1910–1914 to 1990–1994. The estimated coefficients of parameters (perfect collinearity of the age, period, and cohort variables) were obtained by the age-period-cohort analyses with intrinsic estimator method (26). These coefficients were converted to the exponential value [exp (coef.) = e^coef.^], representing the RRs of CVD mortality for a given age, period, or birth cohort relative to the average level of all ages, periods, or birth cohorts combined. Age-period-cohort analysis was performed using STATA 15.0 software (StataCorp, College Station, TX, United States). The Wald's chi-square test was adopted to assess the significance of the estimable parameters and functions. All statistical tests were 2-sided and *P*-values < 0.05 were considered statistically significant.

## 3. Results

### 3.1. Descriptive analysis

**Figure 1:**
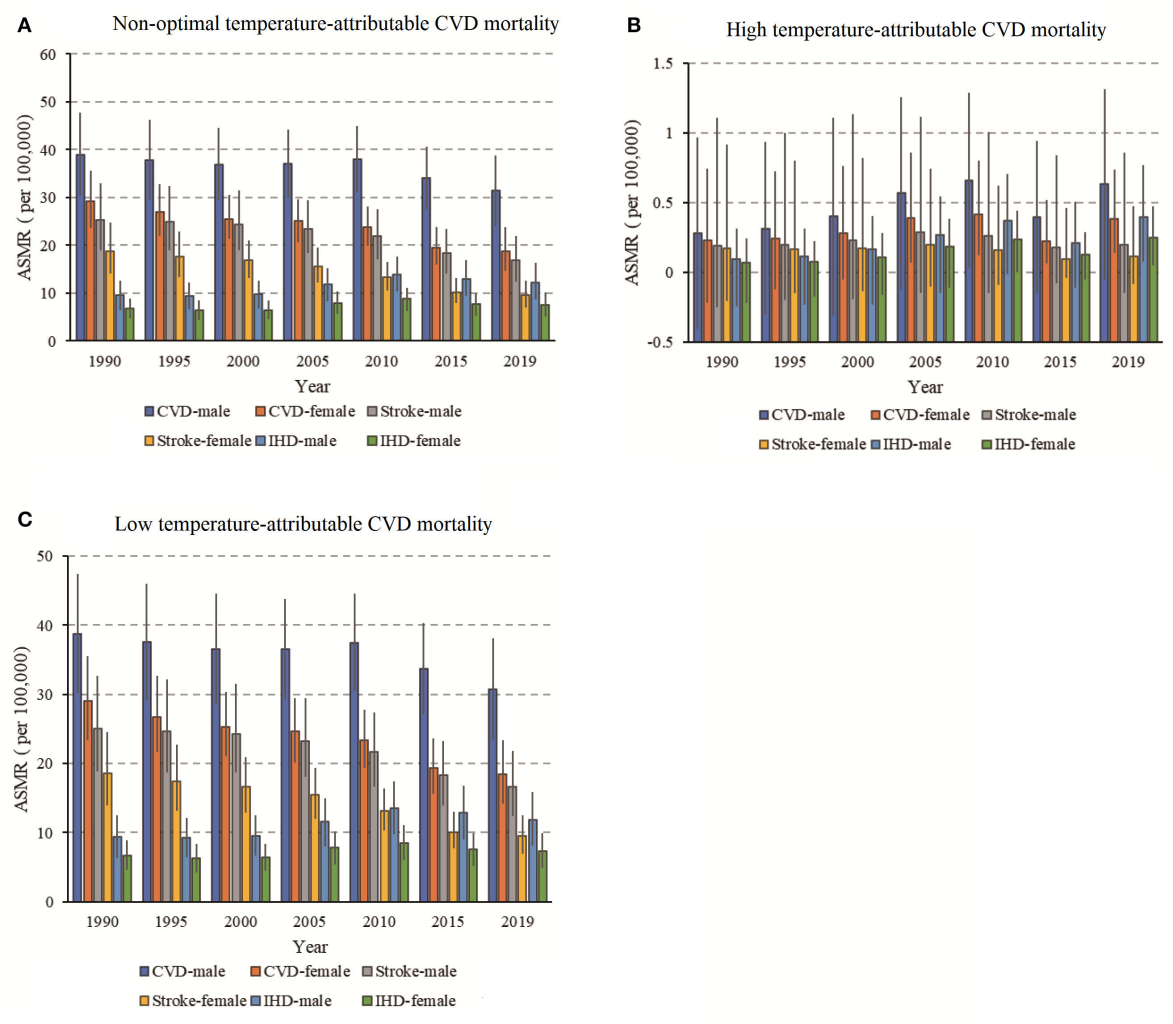
The trends of age-standardized mortality rate (ASMR) for CVD **(A)**, stroke **(B)**, and IHD **(C)** mortality attributable to non-optimal temperatures during 1990–2019 and the corresponding 95% CI.

The mortality of CVD attributable to non-optimal temperature in China from 1990 to 2019 is shown in Figure 1. Deaths due to the non-optimal temperature were dominated by low temperature. Generally, the ASMR of CVD and stroke attributable to non-optimal temperature showed a downward trend in China from 1990 to 2019. However, slight increments were observed in IHD among both sexes. For CVD, stroke, and IHD, the annual ASMR in males were significantly higher than those in females during the observation period. The ASMR of CVD, stroke, and IHD attributable to non-optimal temperature by age group in China in 2019 were shown in Table 1. In 2019, the ASMR of CVD attributable to non-optimal temperature in China were 31.38 (95% UI 24.69 to 38.81) and 18.82 (95% UI 14.59 to 23.76) per 100,000 population of males and females, respectively. The ASMR of stroke attributable to non-optimal temperature was 16.80 (95% UI 12.44 to 21.94) in males and 9.66(95% UI 7.07 to 12.59) in females, and 12.23 (95% UI 8.63 to 16.29) in males and 7.53 (95% UI 5.18 to 10.11) in females for IHD, respectively, per 100,000 population.

**Table 1:** Sex- and age-specific mortality rates of CVD, stroke, and IHD attributable to non-optimal temperature in China in 1990 and 2019 and their average annual percentage changes (AAPC) from 1990 to 2019.

**Categories**	**Males**		**Females**	
	**Rates in 2019, 95% UI (per 100,000 population)**	**AAPC, 95% CI (%,1990–2019)**	**Rates in 2019, 95% UI (per 100,000 population)**	**AAPC, 95% CI (%, 1990–2019)**
**CVD**				
ASMR	31.38 (24.69 to 38.81)	−0.90 (−1.48 to −0.31)^*^	18.82 (14.59 to 23.76)	−1.77 (−2.62 to −0.91)^*^
25–29 years	0.88 (0.66 to 1.11)	−0.82 (−1.65 to 0.02)	0.30 (0.21 to 0.40)	−3.12 (−4.82 to −1.39)^*^
30–34 years	1.81 (1.36 to 2.31)	−0.51 (−1.35 to 0.35)	0.53 (0.38 to 0.72)	−2.85 (−4.18 to −1.51)^*^
35–39 years	3.40 (2.52 to 4.42)	−0.48 (−0.81 to −0.14)^*^	0.96 (0.69 to1.30)	−3.47 (−3.82 to −3.12)^*^
40–44 years	6.11 (4.41 to 8.11)	−0.74 (−1.49 to 0.01)	1.96 (1.41 to 2.64)	−3.47 (−3.81 to −3.14)^*^
45–49 years	8.69 (6.12 to 11.71)	−1.42 (−2.01 to −0.82)^*^	3.31 (2.42 to 4.49)	−3.45 (−4.69 to −2.20)^*^
50–54 years	15.02 (10.82 to 20.15)	−1.88 (−2.21 to −1.55)^*^	6.56 (4.73 to 8.91)	−3.70 (−4.11 to −3.28)^*^
55–59 years	24.40 (17.39 to 32.70)	−1.98 (−2.38 to −1.59)^*^	11.64 (8.46 to 15.71)	−3.37 (−4.20 to −2.54)^*^
60–64 years	42.05 (30.79 to 55.68)	−1.78 (−2.28 to −1.27)^*^	22.25 (16.64 to 29.83)	−3.00 (−3.64 to −2.35)^*^
65–69 years	72.60 (55.42 to 94.96)	−1.74 (−2.40 to −1.07)^*^	43.01 (32.71 to 55.77)	−2.59 (−3.56 to −1.62)^*^
70–74 years	143 (108.07 to 182.22)	−1.43 (−2.15 to −0.70)^*^	89.82 (68.97 to 116.23)	−2.16 (−2.66 to −1.65)^*^
75–79 years	256.45 (196.65 to 327.39)	−1.10 (−1.74 to −0.46)^*^	168.25 (130.76 to 214.87)	−1.80 (−2.31 to −1.30)^*^
80–84 years	495.54 (389.24 to 610.42)	−0.74 (−1.50 to 0.02)	338.79 (262.76 to 426.06)	−1.34 (−2.31 to −0.37)^*^
**Stroke**				
ASMR	16.80 (12.44 to 21.94)	−1.53 (−2.25 to −0.81)^*^	9.66 (7.07 to 12.59)	−2.54 (−3.13 to −1.94)^*^
25–29 years	0.43 (0.29 to 0.59)	−1.27 (−2.05 to −0.49)^*^	0.15 (0.10 to 0.21)	−3.39 (−5.11 to −1.63)^*^
30–34 years	0.88 (0.60 to 1.19)	−0.97 (−1.78 to −0.15)^*^	0.26 (0.18 to 0.37)	−3.26 (−4.58 to −1.91)^*^
35–39 years	1.70 (1.15 to 2.34)	−0.88 (−1.63 to −0.12)^*^	0.52 (0.35 to 0.74)	−3.81 (−4.13 to −3.49)^*^
40–44 years	3.17 (2.10 to 4.38)	−1.33 (−1.70 to −0.95)^*^	1.13 (0.78 to 1.59)	−3.97 (−4.27 to −3.66)^*^
45–49 years	4.69 (3.16 to 6.59)	−1.97 (−2.56 to −1.38)^*^	1.98 (1.35 to 2.81)	−3.92 (−5.16 to −2.67)^*^
50–54 years	8.38 (5.67 to 11.72)	−2.56 (−2.90 to −2.21)^*^	4.09 (2.79 to 5.72)	−4.14 (−4.54 to −3.74)^*^
55–59 years	14.01 (9.43 to 19.53)	−2.51 (−2.86 to −2.15)^*^	7.17 (4.89 to 10.00)	−3.94 (−4.60 to −3.27)^*^
60–64 years	24.99 (17.14 to 35.14)	−2.21 (−3.03 to −1.38)^*^	13.40 (9.29 to 18.77)	−3.56 (−4.06 to −3.06)^*^
65–69 years	44.10 (31.30 to 60.14)	−2.05 (−2.83 to −1.26)^*^	25.71 (18.52 to 35.10)	−2.96 (−3.87 to −2.05)^*^
70–74 years	86.96 (62.40 to 116.97)	−1.80 (−2.40 to −1.19)^*^	52.50 (38.03 to 70.44)	−2.65 (−3.18 to −2.12)^*^
75–79 years	152.44 (110.73 to 203.34)	−1.61 (−2.28 to −0.95)^*^	94.66 (69.16 to 126.01)	−2.44 (−2.93 to −1.95)^*^
80–84 years	268.48 (200.09 to 348.96)	−1.24 (−2.23 to −0.25)^*^	174.61 (128.69 to 227.14)	−2.03 (−2.64 to −1.41)^*^
**IHD**				
ASMR	12.23 (8.63 to 16.29)	0.69 (0.30 to 1.07)^*^	7.53 (5.18 to 10.11)	0.13 (−0.80 to 1.07)
25–29 years	0.42 (0.28 to 0.57)	−0.02 (−0.95 to 0.92)	0.14 (0.08 to 0.20)	−2.53 (−4.16 to −0.87)^*^
30–34 years	0.87 (0.60 to 1.20)	0.26 (−0.68 to 1.20)	0.24 (0.15 to 0.35)	−2.30 (−4.78 to 0.25)
35–39 years	1.59 (1.06 to 2.23)	0.35 (−0.01 to 0.71)	0.40 (0.25 to 0.58)	−2.53 (−2.95 to −2.11)^*^
40–44 years	2.73 (1.79 to 3.86)	0.08 (−0.17 to 0.34)	0.74 (0.46 to 1.09)	−2.31(−2.42 to −2.20)^*^
45–49 years	3.68 (2.32 to 5.23)	−0.30 (−0.99 to 0.41)	1.18 (0.74 to 1.71)	−2.20 (−3.46 to −0.92)^*^
50–54 years	6.02 (3.85 to 8.57)	−0.47 (−0.80 to −0.15)^*^	2.14 (1.34 to 3.10)	−2.24 (−2.68 to −1.80)^*^
55–59 years	9.35 (5.93 to 13.26)	−0.67 (−1.53 to −0.20)	3.86 (2.41 to 5.53)	−1.87 (−2.81 to −0.81)^*^
60–64 years	15.07 (9.75 to 21.42)	−0.50 (−0.98 to −0.01)^*^	7.62 (4.80 to 10.91)	−1.53 (−2.16 to −0.90)^*^
65–69 years	24.62 (16.23 to 34.36)	−0.44 (−1.26 to 0.37)	14.63 (9.47 to 20.34)	−1.01 (−2.16 to 0.15)
70–74 years	47.16 (31.69 to 65.39)	0.13 (−0.42 to 0.68)	30.97 (20.73 to 42.41)	−0.16 (−0.64 to 0.33)
75–79 years	86.43 (59.68 to 117.20)	0.54 (−0.17 to 1.26)	60.19 (40.56 to 82.20)	0.02 (−0.96 to 1.01)
80–84 years	186.98 (130.56 to 248.56)	1.05 (0.24 to 1.86)^*^	133.90 (92.25 to 179.91)	0.78 (−0.18 to 1.75)

^*^Indicated the AAPC was significantly different from zero at the α = 0.05 level.

UI, uncertainty interval; CI, confidence interval; ASMR, age–standardized mortality rates; AAPC, average annual percent change.

**Figure 2:**
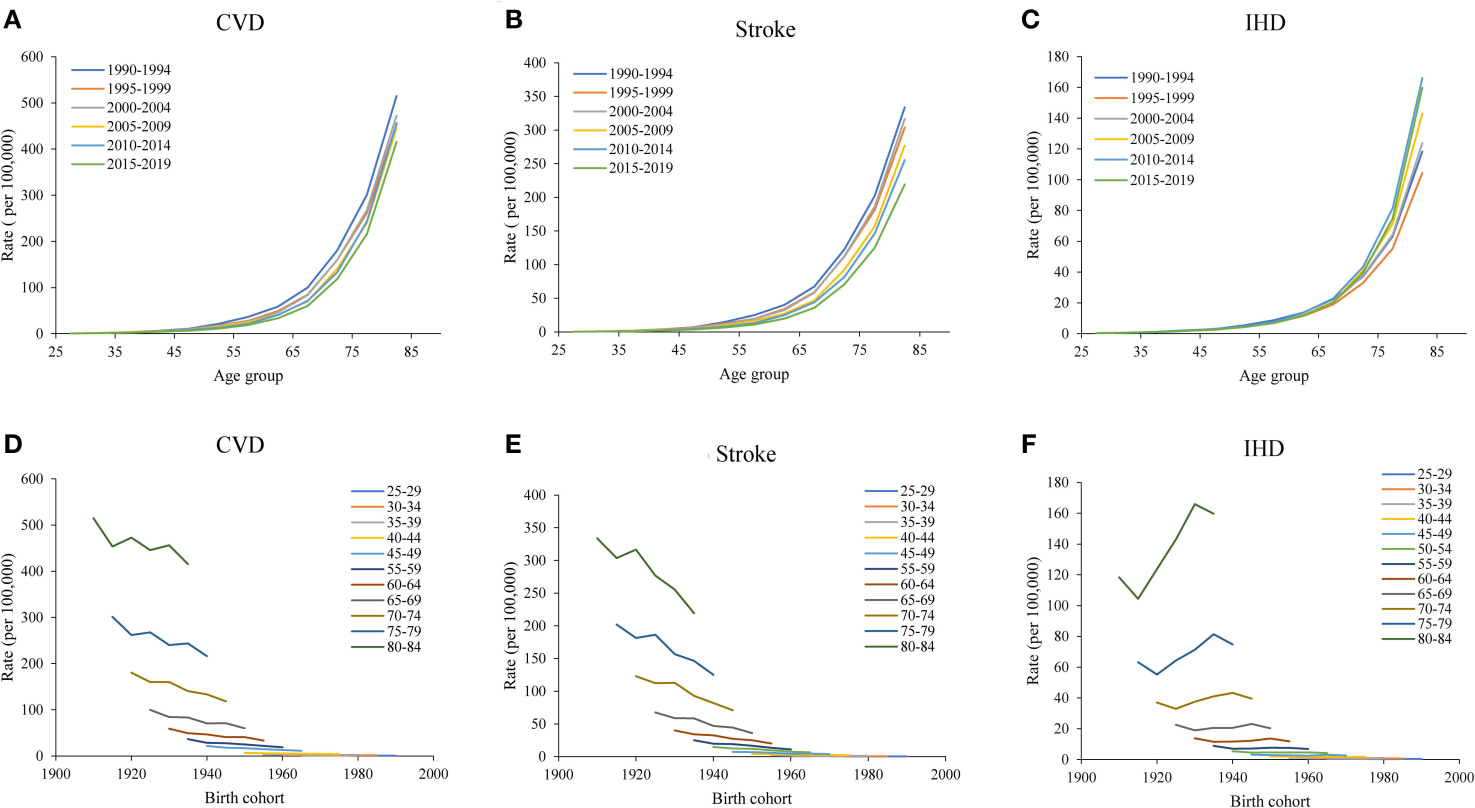
Age-specific mortality of CVD attributable to non-optimal temperatures from 1990 to 2019 and cohort-specific mortality of CVD attributable to non-optimal temperatures from 1990 to 2019. **(A**–**C)** Survey years were arranged into consecutive 6-year periods from 1990 to 1994 (median, 1992), 1995 to 1999 (median, 1997), 2000 to 2004 (median, 2002), 2005 to 2009 (median, 2007), and 2010 to 2014 (median, 2012), and the CVD, stroke, and IHD mortality attributable to non-optimal temperatures increased with age group. **(D**–**F)**, the data of CVD, stroke, and IHD mortality attributable to non-optimal temperatures were arranged into 17 consecutive birth cohorts, including those born from 1910 to 1914 (median, 1912) to 1990 to 1994 (median, 1992), and successive 5-year age intervals from 25 to 29 years (median, 27 years) to 80 to 84 (median, 82 years) years of age.

Figures 2A–C showed the increased mortality of CVD, stroke, and IHD attributable to non-optimal temperature with age group, and the rate accelerated after the group aged 65–69 years. Meanwhile, a declining trend was observed in mortality between 1990 to 1994 and 2015 to 2019. As Figures 2D–F showed, the mortality of CVD and stroke attributable to non-optimal temperature showed a decreased trend across birth cohorts, whereas mortality of IHD first showed a decrease, then an increase, and finally decreased again across all age groups, suggesting a relatively lower risk of mortality in those born later. Because the cohort variation could be confounded by age and period, and unable to assess the net cohort effect. Therefore, age-period-cohort analyses were used to address this limitation.

### 3.2. Joinpoint regression analysis

**Figure 3:**
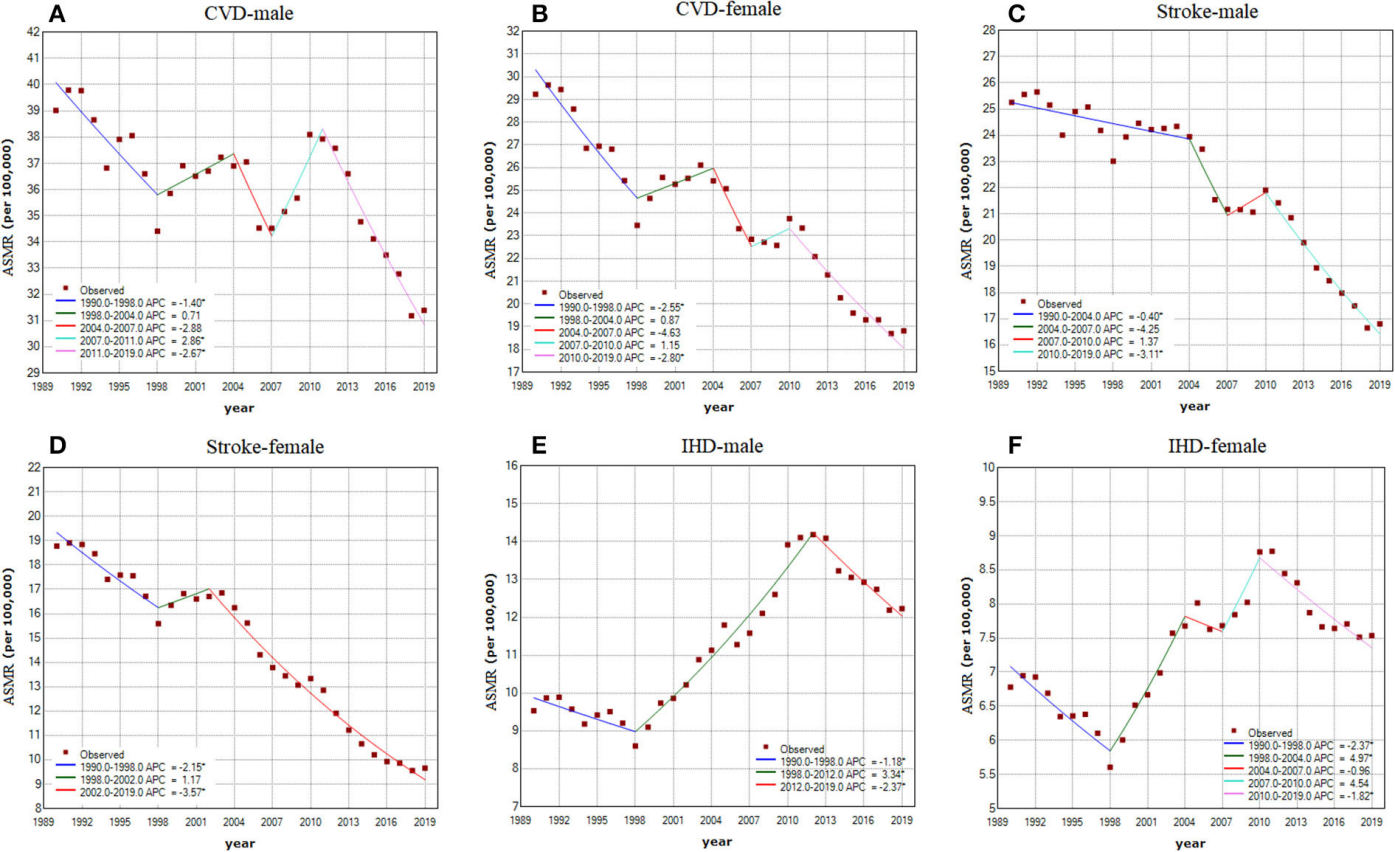
Joinpoint regression analysis in sex-specific age-standardized mortality rate (ASMR) of CVD, stroke, and IHD attributable to non-optimal temperatures from 1990 to 2019. **(A)** CVD in males; **(B)** CVD in females; **(C)** stroke in males; **(D)** stroke in females; **(E)** IHD in males; **(F)** IHD in females. Notes: an asterisk indicates that the annual percent change is statistically significantly different from zero at the α = 0.05 level.

The APC and AAPC by joinpoint regression analysis are listed in Table 1 and Figure 3. From 1990 to 2019, the ASMR of CVD attributable to non-optimal temperature in China decreased by 0.90% (95% CI 0.31–1.48, Figure 3A) in males and 1.77% (95% CI 0.91–2.62, Figure 3B) in females. The ASMR of stroke decreased by 1.53% (95% CI 0.81–2.25) in males (Figure 3C) and 2.54% (95% CI 1.94–3.13) in females (Figure 3D), whereas the ASMR of IHD rose by 0.69% (95% CI 0.30–1.07) in males (Figure 3E) and 0.13% (−0.80 to 1.07) in females (Figure 3F). Moreover, there were marked sex differences in the AAPC of CVD, stroke, and IHD across all age groups, with less improvement in mortality in males than in females.

### 3.3. Age-period-cohort analysis

**Figure 4:**
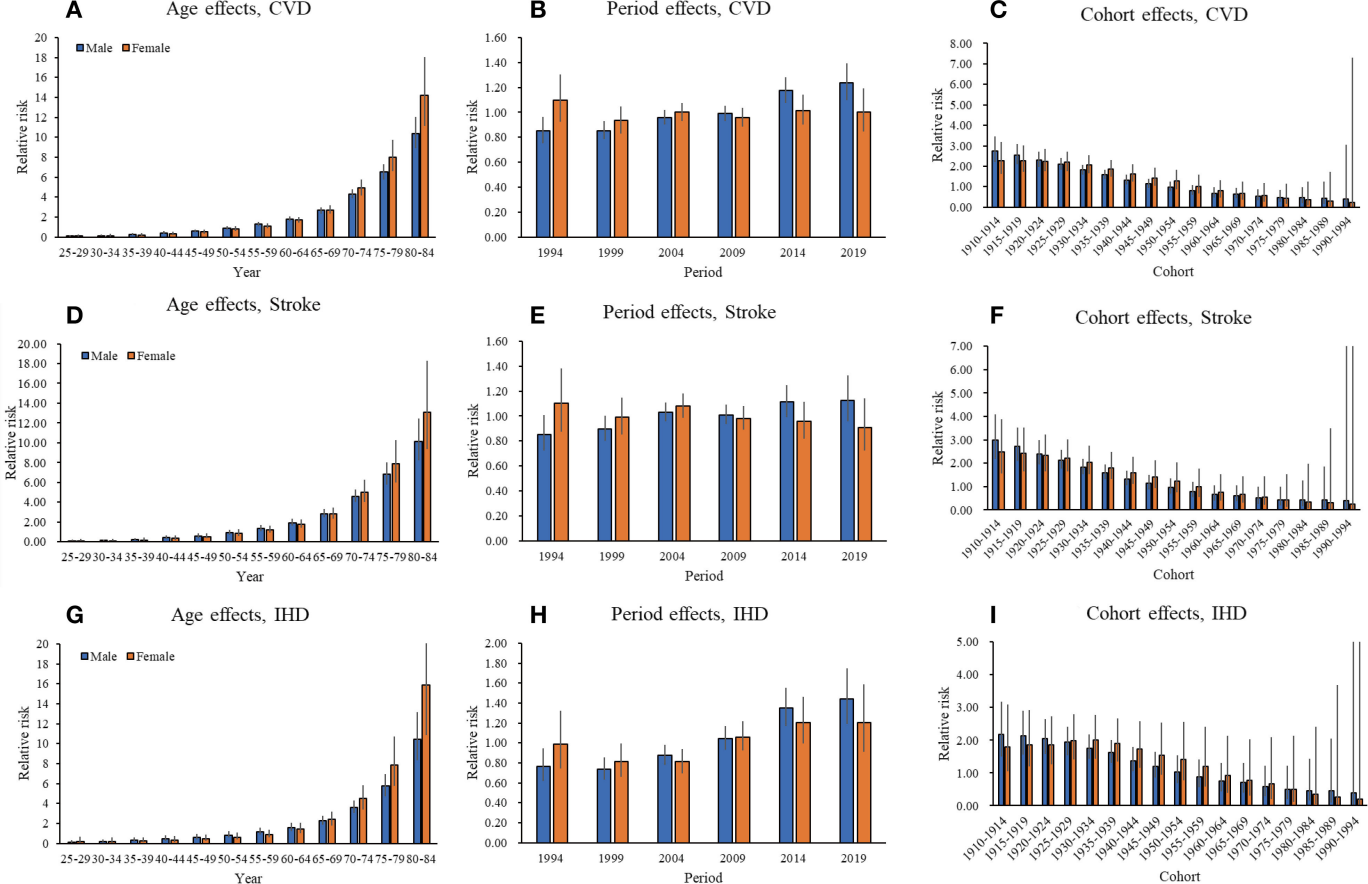
Parameter estimates of age, period, and cohort effects on CVD, stroke, and IHD mortality attributable to non-optimal temperatures from 1990 to 2019. **(A**, **D**, **G)** Age relative risk of CVD, stroke, and IHD mortality attributable to non-optimal temperatures and the corresponding 95% CI. **(B**, **E**, **H)** Period relative risks of CVD, stroke, and IHD mortality attributable to non-optimal temperatures and the corresponding 95% CI. The period relative risk was adjusted for age and nonlinear cohort effects. **(C**, **F**, **I)** Cohort relative risks of CVD, stroke, and IHD mortality attributable to non-optimal temperatures and the corresponding 95% CI. The cohort relative risk was adjusted for age and nonlinear period effects.

The estimated RR of age, period, and cohort effects of CVD mortality attributable to non-optimal temperature for both sexes were shown in Table 2 and Figure 4. Age effects of CVD mortality showed an expected exponential distribution for both sexes in China. After adjustment for period and cohort deviations, the age effect on CVD mortality increased from 0.08 (95% CI 0.04–0.17) in the group aged 25–29 to 10.35 (95% CI 8.92–12.01) in the group aged 80–84 for males, and from 0.10 (95% CI 0.04–0.26) in the group aged 25–29 to 14.18 (95% CI 11.12–18.08) in the group aged 80–84 for females (Figure 4A). The period effects of the mortality risk showed a slight increase from 0.85 (95% CI 0.75–0.96) in 1990 to 1.24 (95% CI 1.10–1.39) in 2019 for males, whereas the period effects were flat for females, indicating no improvements for the whole population during the study period (Figure 4B). The cohort effects for both sexes continuously decreased, which means the later birth cohorts experienced a relatively lower mortality risk compared to the earlier cohorts (Figure 4C).

**Table 2:** Sex-specific relative risks of CVD death attributable to non-optimal temperatures in China due to age, period, and cohort effects.

**Factor**	**Mortality in males**		**Mortality in females**	
	**RR (95% CI)**	***P*** **-value**	**RR (95% CI)**	***P*** **-value**
**Age**				
25–29	0.08 (0.04–0.17)	< 0.001	0.10 (0.04–0.26)	< 0.001
30–34	0.14 (0.08–0.24)	< 0.001	0.14 (0.07–0.29)	< 0.001
35–39	0.24 (0.16–0.37)	< 0.001	0.20 (0.11–0.36)	< 0.001
40–44	0.42 (0.30–0.59)	< 0.001	0.33 (0.21–0.53)	< 0.001
45–49	0.58 (0.43–0.77)	< 0.001	0.50 (0.34–0.72)	< 0.001
50–54	0.89 (0.71–1.12)	0.310	0.80 (0.60–1.06)	0.120
55–59	1.28 (1.06–1.53)	0.009	1.10 (0.87–1.38)	0.440
60–64	1.81 (1.57–2.09)	< 0.001	1.69 (1.41–2.03)	< 0.001
65–69	2.66 (2.37–2.99)	< 0.001	2.71 (2.31–3.17)	< 0.001
70–74	4.31 (3.87–4.80)	< 0.001	4.93 (4.19–5.79)	< 0.001
75–79	6.50 (5.76–7.33)	< 0.001	7.99 (6.57–9.72)	< 0.001
80–84	10.35 (8.92–12.01)	< 0.001	14.18 (11.12–18.08)	< 0.001
**Period**				
1990–1994	0.85 (0.75–0.96)	0.011	1.10 (0.92–1.30)	0.292
1995–1999	0.85 (0.78–0.93)	< 0.001	0.93 (0.83–1.05)	0.242
2000–2004	0.96 (0.90–1.02)	0.174	1.00 (0.93–1.08)	0.980
2005–2009	0.99 (0.93–1.05)	0.741	0.96 (0.89–1.03)	0.270
2010–2014	1.18 (1.08–1.28)	< 0.001	1.01 (0.90–1.14)	0.813
2015–2019	1.24 (1.10–1.39)	0.001	1.00 (0.85–1.19)	0.961
**Cohort**				
1910–1914	2.75 (2.18–3.47)	< 0.001	2.27 (1.63–3.17)	< 0.001
1915–1919	2.56 (2.11–3.09)	< 0.001	2.27 (1.71–3.01)	< 0.001
1920–1924	2.31 (1.97–2.71)	< 0.001	2.23 (1.75–2.84)	< 0.001
1925–1929	2.08 (1.81–2.39)	< 0.001	2.19 (1.76–2.72)	< 0.001
1930–1934	1.82 (1.59–2.08)	< 0.001	2.05 (1.66–2.54)	< 0.001
1935–1939	1.60 (1.40–1.84)	< 0.001	1.85 (1.48–2.31)	< 0.001
1940–1944	1.33 (1.13–1.57)	< 0.001	1.61 (1.25–2.09)	< 0.001
1945–1949	1.14 (0.93–1.40)	0.205	1.43 (1.05–1.94)	0.024
1950–1954	0.97 (0.76–1.25)	0.834	1.27 (0.88–1.83)	0.198
1955–1959	0.81 (0.61–1.09)	0.173	1.02 (0.66–1.58)	0.922
1960–1964	0.68 (0.48–0.96)	0.030	0.80 (0.48–1.33)	0.383
1965–1969	0.64 (0.43–0.95)	0.027	0.69 (0.38–1.23)	0.208
1970–1974	0.54 (0.33–0.87)	0.011	0.57 (0.28–1.17)	0.127
1975–1979	0.46 (0.26–0.84)	0.011	0.45 (0.18–1.13)	0.090
1980–1984	0.45 (0.21–0.96)	0.040	0.35 (0.10–1.26)	0.110
1985–1989	0.44 (0.16–1.25)	0.124	0.30 (0.05–1.72)	0.177
1990–1994	0.39 (0.05–3.06)	0.372	0.24 (0.01–7.32)	0.411
***P*** **trend**	< 0.001		< 0.001	
Deviance	0.33		2.43	
AIC	3.32		5.47	
BIC	−170.74		−168.64	

RR denotes the relative risk of CVD death in particular age, period, or birth cohort relative to the average level of all ages, periods, or birth cohorts combined.

RR, relative risk; CI, confidence interval; AIC, Akaike Information Criterion; BIC, Bayesian Information Criterion.

The estimated RR of age, period, and cohort effects of stroke mortality attributable to non-optimal temperature for both sexes were shown in Supplementary Table 1. With regard to stroke, the mortality risk also increased markedly with age, regardless of sex (Figure 4D). Periods have a non-significant effect on stroke mortality attributable to non-optimal temperature. Estimated period effects showed an upward trend in males during the entire period. In contrast, the females have seen a slight improvement in stroke mortality (Figure 4E). The cohort effects for both sexes also showed downward trends, which were similar to CVD (Figure 4F).

The estimated RR of age, period, and cohort effects of IHD mortality attributable to non-optimal temperature for both sexes were shown in Supplementary Table 2. The mortality risk caused by IHD attributable to non-optimal temperature increased markedly with advancing age for both sexes (Figure 4G). Period effects were greater for IHD mortality than on stroke across the study period (Figures 4E, H). The period effects of the mortality risk caused by IHD for both sexes showed an upward trend between 1990 and 2019. It increased from 0.77 (95% CI 0.62–0.94) in 1990 to 1.44 (95% CI 1.19–1.75) in 2019 for males, and from 0.99 (95% CI 0.74–1.32) in 1990 to 1.20 (95% CI 0.91–1.59) in 2019 for females. For cohort effects, it showed downward trends for both sexes, which were similar to CVD and stroke (Figure 4I).

## 4. Discussion

This study comprehensively estimated on temporal trends in CVD deaths attributable to non-optimal temperature from 1990 to 2019 in China. Although a downward trend of the ASMR was observed for CVD mortality attributable to non-optimal temperature among males and females, the death burden remains substantial, with stroke being the main burden of temperature-related deaths from CVD. In addition, we found that the mortality of IHD caused by non-optimal temperature showed an upward trend. Over the past 30 years, the CVD death burden attributable to low temperature was higher than the high temperature, but it showed an increasing trend attributable to high temperature.

Previous studies had generally found that the death burden caused by low temperatures was higher than that of high temperatures (12–14, 16, 27). A study in China showed that 8.86% of CVD deaths were caused by low temperatures, and 0.17% were caused by high temperatures (16). Another study including 272 main Chinese cities reported that the PAFs attributable to low and high temperatures were 11.62 and 2.71%, respectively (14). Our results are consistent with previous studies. There are several possible reasons for this phenomenon. First, the duration of cold waves is typically greater than that of hot waves, which can lead to longer exposure to low temperatures than to high temperatures (28). Second, to date, the overall increase in ambient temperature is not very large, and the annual mean temperature increases slowly so that the CVD death burden due to high temperatures is relatively small. However, the global temperature has increased by about 1.25°C over the 20th century and the current emissions trajectory suggests that it will exceed 1.5°C in < 10 years (29). It means that future global warming could exacerbate the adverse health effects of high temperature and increase the burden of disease caused by high temperature. In addition, one study showed a higher risk of myocardial infarction as a consequence of heat exposure compared to cold exposure (30, 31). This may explain why the IHD death burden attributable to high temperature has generally shown an increased trend among both sexes since 1990. Overall, we should not only focus on the impact of high temperatures on CVD under unavoidable global warming, but also develop a better plan to reduce the CVD death burden due to low temperatures.

We also found there were clear gender differences in CVD death burden attributable to non-optimum temperatures, which was higher in males regardless of high or low temperatures. In addition, we can observe that the AAPC mortality of CVD for females decreased greater than for males across all age groups from 1990 to 2019 in Table 1. Previous studies have shown gender differences in mortality risk patterns of CVD caused by non-optimal temperature; similar between males and females (32), higher in females (33), higher in males (34). The underlying reasons for these gender differences remain unclear and should be explored in future prospective studies. Some researchers argued that these differences may be related to factors such as thermoregulation, physiological responses, culture, and socioeconomic (35, 36). For example, influenced by economic structure and culture in China, men generally engage in more outdoor activities and physical labor in high or low temperature environments, while women tend to engage in light labor indoors. To some extent, this may explain the differences between males and females that we observed in our analysis.

The results of the age-period-cohort effect analysis concluded that age was a risk factor for CVD death, including IHD and stroke, attributable to non-optimal temperature. Age effect showed continuously increasing with age among males and females. Similar to our findings, most previous studies consistently showed that the elderly are more vulnerable to the effects of temperature than younger populations (14, 33, 35, 37). This may be ascribed to senescence, which leads to impairments of thermoregulatory capacity and degeneration of physiologic functions, as well as the possibility of the existence of multiple chronic diseases (31). The findings suggest that as the population age, governments and health authorities should focus on these elderly populations and develop targeted measures to protect them from the effects of non-optimal temperature. For example, strengthening the prediction and monitoring of early cold and hot spells, timely issuing warnings to the public; giving full play to the role of community health, popularizing the knowledge of preventing non-optimal temperature to residents, and transforming the knowledge into behavioral adaptation.

The period effects showed an increasing trend in males CVD death, including stroke, attributable to non-optimal temperature across 30 years, whereas it was flat in females. The risk of death for IHD rises the most strikingly for both sexes in period effects. Among all environmental risk factors attributable to CVD mortality, the ranking and value of ASMR due to non-optimal temperature in stroke decreased, while increasing in IHD (Supplementary Figures 1–3). In addition, the ranking of IHD attributed to other risks also rose, such as poor diet, and an increasing prevalence of hypertension. The decline in stroke mortality was the main cause of the improvement in CVD mortality, largely offsetting the increases in IHD mortality. Previous studies reported that out-of-hospital IHD mortality in China was high, with only 11% receiving basic cardiopulmonary resuscitation when IHD occurred, reflecting inadequate knowledge of health first aid (21, 38). The irregularities of guideline-recommended treatments (e.g., β-blockers and angiotensin-converting enzyme inhibitors) remained common and had not significantly improved, which may also contribute to a lack of improvements over time in IHD mortality (39).

The cohort effects showed continuously decreasing trends as a whole, and the cohorts born later had lower mortality risk. This may be relevant to improvements in healthcare coverage, upgrades in early diagnosis of the disease, and advances in treatment techniques and disease management, as well as improvements in public health initiatives in CVD prevention. However, previous studies reported that CVD risk factors were more prevalent in the later birth cohort than in the earlier birth cohort such as air pollution, traffic noise, and bad urban city planning (4, 31, 40). In addition, modifiable risk factors for CVD death are increasing, including high BMI, hypertension, hypercholesterolemia, and poor diet, which are substantial gaps between recommended goals and warrant increased policy and health system attention (21).

This study has some advantages. To the best of our knowledge, this is the first study to investigate the temporal trends of CVD mortality attributable to non-optimal temperatures in China. Besides, data of the GBD 2019 uses the unified and standard methodology to provide consistent estimates of age- and sex-specific all-cause and cause-specific mortality for 369 diseases in 204 countries and territories, which could reduce the potential for misclassification of results and are comparable across time. Moreover, both alterations over the entire period (assessed by the AAPC) and each segmental period (assessed by the APC) were determined using a joinpoint regression model. Furthermore, the effects of age, period, and birth cohort were explored, allowing for the analysis of particular time periods on non-optimal temperature contribution to CVD mortality rather than the risk carried by the birth cohort. Lastly, the main clinical implications of this study were that patients with CVDs should be advised to minimize exposure to non-optimal temperatures and to enhance care for the target population, especially the elderly.

Our study has several limitations. First, the data of CVD death attributable to non-optimum temperatures was only an estimate, and temperature effects were defined as short-term effects that occur on the day of exposure and did not consider the lagged and cumulative effects, which may underestimate the burden of CVD associated with non-optimum temperatures. Second, there may be ecological fallacies because temperature exposure was assessed using ambient temperature and not based on individual-level exposure. Thirdly, due to the unavailability of data, we could not consider the effects of socioeconomic status, air conditioning and heating usage, and development levels of infrastructure and public health services on CVD mortality, which also may underestimate the effects of non-optimum temperatures on CVD death. Finally, the age-period-cohort analysis, which was based on cross-sectional GBD data from 1990 to 2019 rather than a cohort study, is subject to an ecological fallacy, since the interpretation of findings at the population level may not hold up at the individual level. Therefore, Large cohort studies are needed to confirm these findings in the future. Despite the limitations, the results presented here provide valuable information for public health policy by highlighting the secular trend in deaths attributable to CVD from non-optimum temperatures compared with other risk factors. It suggests that efforts to address vulnerability should support, focus on target populations (e. g., older people) and potential disease burden (e. g., IHD), or develop strategies to reduce exposure (e. g., housing insulation, air conditioning) or health education.

## 5. Conclusion

Although there have been reductions in CVD mortality attributable to non-optimum temperatures during the past 30 years, the mortality of IHD has increased in China, which indicates the burden remains high. Low temperatures have a greater death burden than high temperatures, but the death burden from high temperatures showed an increasing trend. With a reduction in CVD mortality attributable to non-optimum temperatures across all age groups over time, but generally, the decreases were smaller in men than in women and the death burden in men was greater than in women. In addition, although the cohorts born later had lower mortality risk, age and period effects showed unfavorable trends. In the context of global climate change, our results call for more attention and strategies to address climate change that protect human health from non-optimal temperatures.

## Data availability statement

The original contributions presented in the study are included in the article/Supplementary material, further inquiries can be directed to the corresponding authors.

## Author contributions

LC, TW, and JW contributed to the conception and study design, revised the article critically for important intellectual content, and interpreted the results. JW, PW, FX, JM, XZ, ZY, and ZG contributed to acquisition of data, analysis, and interpretation of data. JW drafted the manuscript. All authors read and approved the final manuscript.

## References

[1] Mensah G Roth G Fuster V. The global burden of cardiovascular diseases and risk factors: 2020 and beyond. J Am Coll Cardiol. (2019) 74:2529–32. 10.1016/j.jacc.2019.10.009 31727292

[2] GBD 2019 Diseases and Injuries Collaborators. Global burden of 369 diseases and injuries in 204 countries and territories, 1990–2019: a systematic analysis for the Global Burden of Disease Study 2019. Lancet. (2020) 396:1204–22. 10.1016/S0140-6736(20)30925-9 33069326 PMC7567026

[3] Yusuf S Joseph P Rangarajan S Islam S Mente A Hystad P. Modifiable risk factors, cardiovascular disease, and mortality in 155 722 individuals from 21 high-income, middle-income, and low-income countries (PURE): a prospective cohort study. Lancet. (2020) 395:795–808. 10.1016/S0140-6736(19)32008-2 31492503 PMC8006904

[4] Roth GA Mensah GA Johnson CO Addolorato G Ammirati E Baddour LM. Global burden of cardiovascular diseases and risk factors, 1990–2019: update from the GBD 2019 study. J Am Coll Cardiol. (2020) 76:2982–3021. 10.1016/j.jacc.2020.11.010 33309175 PMC7755038

[5] Zhou M Wang H Zeng X Yin P Zhu J Chen W. Mortality, morbidity, and risk factors in China and its provinces, 1990-2017: a systematic analysis for the Global Burden of Disease Study 2017. Lancet. (2019) 394:1145–58. 10.1016/S0140-6736(19)30427-1 31248666 PMC6891889

[6] Li S Liu Z Joseph P Hu B Yin L Tse LA. Modifiable risk factors associated with cardiovascular disease and mortality in China: a PURE substudy. Eur Heart J. (2022) 43:2852–63. 10.1093/eurheartj/ehac268 35731140

[7] Lv J Yu C Guo Y Bian Z Yang L Chen Y. Adherence to healthy lifestyle and cardiovascular diseases in the Chinese population. J Am Coll Cardiol. (2017) 69:1116–25. 10.1016/j.jacc.2016.11.076 28254173 PMC6675601

[8] Wang T Zhao Z Yu X Zeng T Xu M Xu Y. Age-specific modifiable risk factor profiles for cardiovascular disease and all-cause mortality: a nationwide, population-based, prospective cohort study. Lancet Regional Health Western Pacific. (2021) 17:100277. 10.1016/j.lanwpc.2021.100277 35005664 PMC8720788

[9] Li Y Wang DD Ley SH Howard AG He Y Lu Y. Potential impact of time trend of life-style factors on cardiovascular disease burden in China. J Am Coll Cardiol. (2016) 68:818–33. 10.1016/j.jacc.2016.06.011 27539174 PMC5850940

[10] Romanello M McGushin A Di Napoli C Drummond P Hughes N Jamart L. The 2021 report of the Lancet Countdown on health and climate change: code red for a healthy future. Lancet. (2021) 398:1619–62. 10.1016/S0140-6736(21)01787-6 34687662 PMC7616807

[11] GBD 2019 Risk Factors Collaborators. Global burden of 87 risk factors in 204 countries and territories, 1990-2019: a systematic analysis for the Global Burden of Disease Study 2019. Lancet. (2020) 396:1223–49. 10.1016/S0140-6736(20)30752-2 33069327 PMC7566194

[12] Burkart KG Brauer M Aravkin AY Godwin WW Hay SI He J. Estimating the cause-specific relative risks of non-optimal temperature on daily mortality: a two-part modelling approach applied to the Global Burden of Disease Study. Lancet. (2021) 398:685–97. 10.1016/S0140-6736(21)01700-1 34419204 PMC8387975

[13] Gasparrini A Guo Y Hashizume M Lavigne E Zanobetti A Schwartz J. Mortality risk attributable to high and low ambient temperature: a multicountry observational study. Lancet. (2015) 386:369–75. 10.1016/S0140-6736(14)62114-0 26003380 PMC4521077

[14] Chen R Yin P Wang L Liu C Niu Y Wang W. Association between ambient temperature and mortality risk and burden: time series study in 272 main Chinese cities. BMJ. (2018) 363:k4306. 10.1136/bmj.k4306 30381293 PMC6207921

[15] Achebak H Devolder D Ballester J. Trends in temperature-related age-specific and sex-specific mortality from cardiovascular diseases in Spain: a national time-series analysis. Lancet Planetary Health. (2019) 3:e297–306. 10.1016/S2542-5196(19)30090-7 31230996

[16] Liu J Liu T Burkart KG Wang H He G Hu J. Mortality burden attributable to high and low ambient temperatures in China and its provinces: results from the Global Burden of Disease Study 2019. Lancet Regional Health Western Pacific. (2022) 24:100493. 10.1016/j.lanwpc.2022.100493 35756888 PMC9213765

[17] The State Council of the People's Republic of China State Council issues plan to prevent chronic diseases. Available online at: http://english.gov.cn/policies/latest_releases/2017/02/14/content_281475567482818.htm (accessed August 21, 2018).

[18] Yang L Li L Lewington S Guo Y Sherliker P Bian Z. Outdoor temperature, blood pressure, and cardiovascular disease mortality among 23 000 individuals with diagnosed cardiovascular diseases from China. Eur Heart J. (2015) 36:1178–85. 10.1093/eurheartj/ehv023 25690792 PMC4430682

[19] Tian Y Liu H Si Y Cao Y Song J Li M. Association between temperature variability and daily hospital admissions for cause-specific cardiovascular disease in urban China: a national time-series study. PLoS Med. (2019) 16:e1002738. 10.1371/journal.pmed.1002738 30689640 PMC6349307

[20] Kang Y Tang H Zhang L Wang S Wang X Chen Z. Long-term temperature variability and the incidence of cardiovascular diseases: a large, representative cohort study in China. Environ Pollut. (2021) 278:116831. 10.1016/j.envpol.2021.116831 33711625

[21] Zhao D Liu J Wang M Zhang X Zhou M. Epidemiology of cardiovascular disease in China: current features and implications. Nat Rev Cardiol. (2019) 16:203–12. 10.1038/s41569-018-0119-4 30467329

[22] Yin Q Wang J Ren Z Li J Guo Y. Mapping the increased minimum mortality temperatures in the context of global climate change. Nat Commun. (2019) 10:4640. 10.1038/s41467-019-12663-y 31604931 PMC6789034

[23] GBD 2017 Causes of Death Collaborators. Global, regional, and national age-sex-specific mortality for 282 causes of death in 195 countries and territories, 1980–2017: a systematic analysis for the Global Burden of Disease Study 2017. Lancet. (2018) 392:1736–88. 10.1016/S0140-6736(18)32203-7 30496103 PMC6227606

[24] Zou Z Cini K Dong B Ma Y Ma J Burgner DP. Time trends in cardiovascular disease mortality across the BRICS: an age-period-cohort analysis of key nations with emerging economies using the global burden of disease study 2017. Circulation. (2020) 141:790–9. 10.1161/CIRCULATIONAHA.119.042864 31941371

[25] Clayton D Schifflers E. Models for temporal variation in cancer rates. II: Age-period-cohort models. Stat Med. (1987) 6:469–81. 10.1002/sim.4780060406 3629048

[26] Yang Y Land KC. Age-Period-Cohort Analysis: New Models, Methods, and Empirical Applications (1st ed.). London: Chapman and Hall/CRC (2013).

[27] Chen S Xiao Y Zhou M Zhou C Yu M Huang B. Comparison of life loss per death attributable to ambient temperature among various development regions: a nationwide study in 364 locations in China. Environ Health. (2020) 19:98. 10.1186/s12940-020-00653-3 32933549 PMC7491140

[28] Guo Y Gasparrini A Armstrong B Li S Tawatsupa B Tobias A. Global variation in the effects of ambient temperature on mortality: a systematic evaluation. Epidemiology. (2014) 25:781–9. 10.1097/EDE.0000000000000165 25166878 PMC4180721

[29] Matthews H Wynes S. Current global efforts are insufficient to limit warming to 1.5°C. Science. (2022) 376:1404–9. 10.1126/science.abo3378 35737785

[30] Bhaskaran K Armstrong B Hajat S Haines A Wilkinson P Smeeth L. Heat and risk of myocardial infarction: hourly level case-crossover analysis of MINAP database. BMJ. (2012) 345:e8050. 10.1136/bmj.e8050 23243290 PMC3521646

[31] Munzel T Hahad O Sorensen M Lelieveld J Duerr GD Nieuwenhuijsen M. Environmental risk factors and cardiovascular diseases: a comprehensive review. Cardiovasc Res. (2021). 10.1093/cvr/cvab316 34609502 PMC9648835

[32] Basu R Ostro B. A multicounty analysis identifying the populations vulnerable to mortality associated with high ambient temperature in California. Am J Epidemiol. (2008) 168:632–7. 10.1093/aje/kwn170 18663214

[33] Son J Lee J Anderson G Bell M. Vulnerability to temperature-related mortality in Seoul, Korea. Environ Res Lett. (2011) 6:034027. 10.1088/1748-9326/6/3/034027 23335945 PMC3546816

[34] Ban J Xu D He MZ Sun Q Chen C Wang W. The effect of high temperature on cause-specific mortality: a multi-county analysis in China. Environ Int. (2017) 106:19–26. 10.1016/j.envint.2017.05.019 28554097 PMC5760246

[35] Hajat S Kovats R Lachowycz K. Heat-related and cold-related deaths in England and Wales: who is at risk? Occup Environ Med. (2007) 64:93–100. 10.1136/oem.2006.029017 16990293 PMC2078436

[36] Alahmad B Shakarchi AF Khraishah H Alseaidan M Gasana J Al-Hemoud A. Extreme temperatures and mortality in Kuwait: Who is vulnerable? Sci Total Environ. (2020) 732:139289. 10.1016/j.scitotenv.2020.139289 32438154

[37] Tian Z Li S Zhang J Jaakkola J Guo Y. Ambient temperature and coronary heart disease mortality in Beijing, China: a time series study. Environ Health. (2012) 11:56. 10.1186/1476-069X-11-56 22909034 PMC3490736

[38] Shao F Li C Liang L Li D Ma S. Outcome of out-of-hospital cardiac arrests in Beijing, China. Resuscitation. (2014) 85:1411–7. 10.1016/j.resuscitation.2014.08.008 25151546

[39] Li J Li X Wang Q Hu S Wang Y Masoudi FA. ST-segment elevation myocardial infarction in China from 2001 to 2011 (the China PEACE-Retrospective Acute Myocardial Infarction Study): a retrospective analysis of hospital data. Lancet. (2015) 385:441–51. 10.1016/S0140-6736(14)60921-1 24969506 PMC4415374

[40] Schrijvers E Verhaaren B Koudstaal P Hofman A Ikram M Breteler M. Is dementia incidence declining?: Trends in dementia incidence since 1990 in the Rotterdam Study. Neurology. (2012) 78:1456–63. 10.1212/WNL.0b013e3182553be6 22551732

