# Supplementary material for: Time trends in cardiovascular disease mortality attributable to non-optimal temperatures in China: An age-period-cohort analysis using the Global Burden of Disease Study 2019
Source: Front Public Health. 2023 Apr 5;10:1075551. doi: 10.3389/fpubh.2022.1075551 (PMC10113563; doi:10.3389/fpubh.2022.1075551)
Supplement: Supplementary file 1 [file Data_Sheet_1.docx]

**Supplementary Material**

**Supplementary Figures**


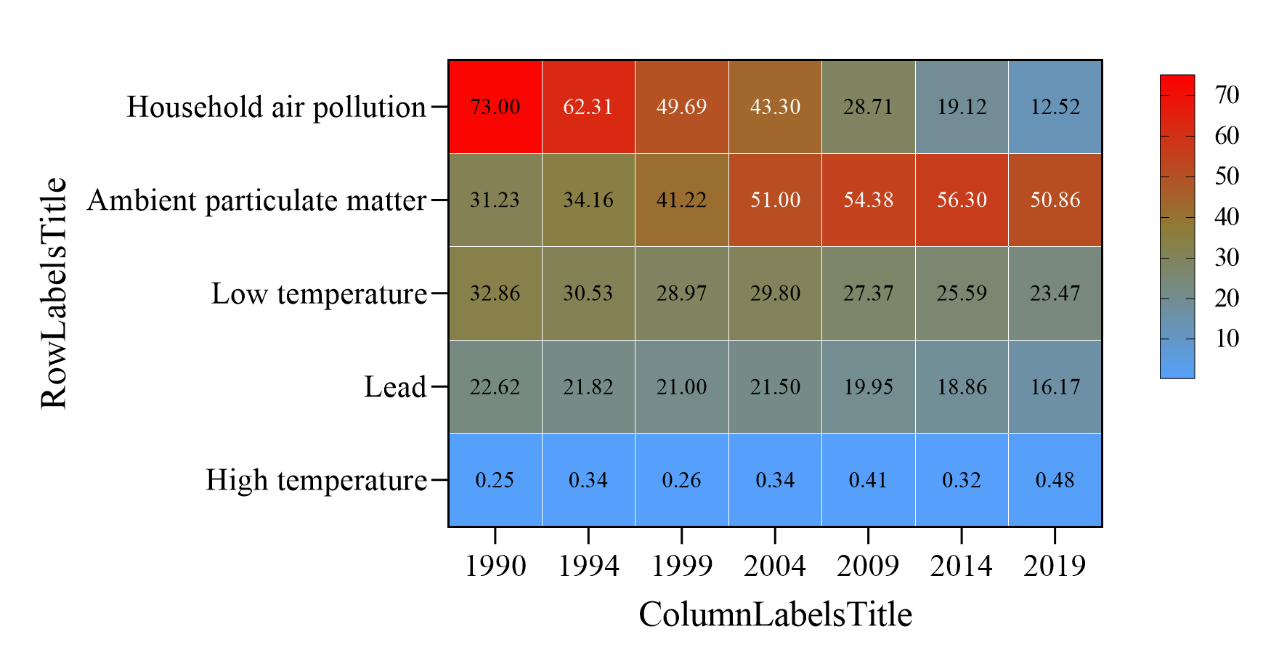


Fig. S1 The age-standardized mortality of CVD due to environmental risk factors in China in 1990, 1994, 1999, 2004, 2009, 2014, and 2019.


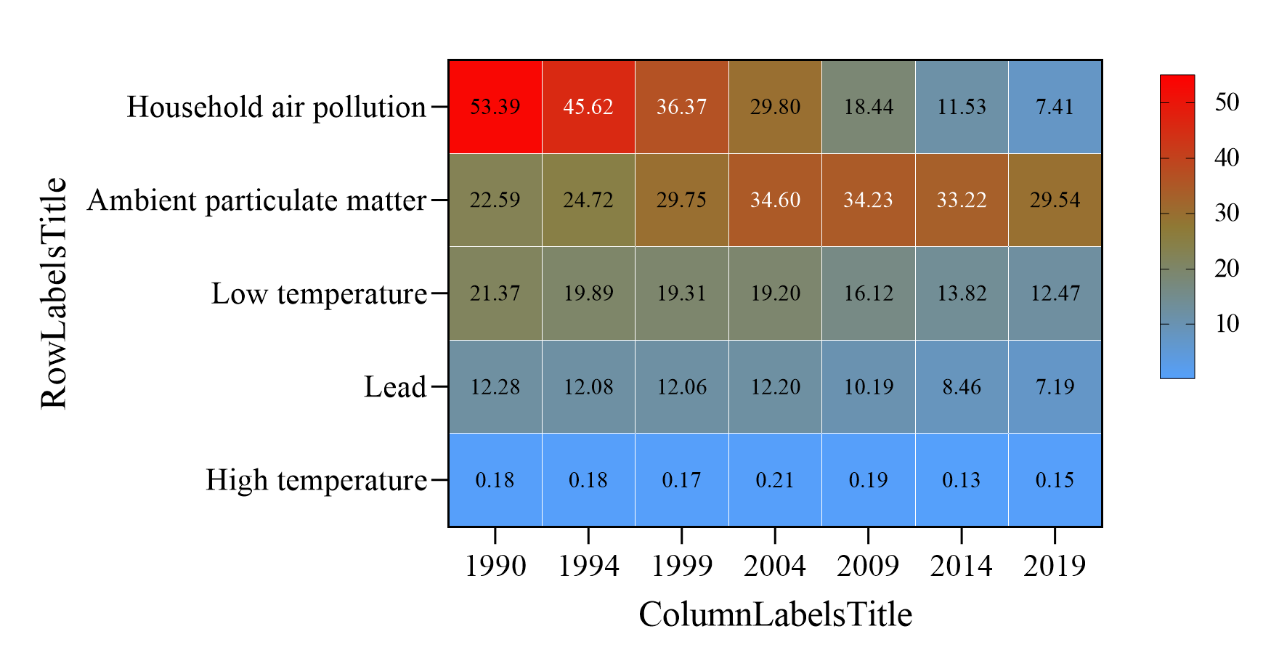


Fig. S2 The age-standardized mortality of stroke due to environmental risk factors in China in 1990, 1994, 1999, 2004, 2009, 2014, and 2019.


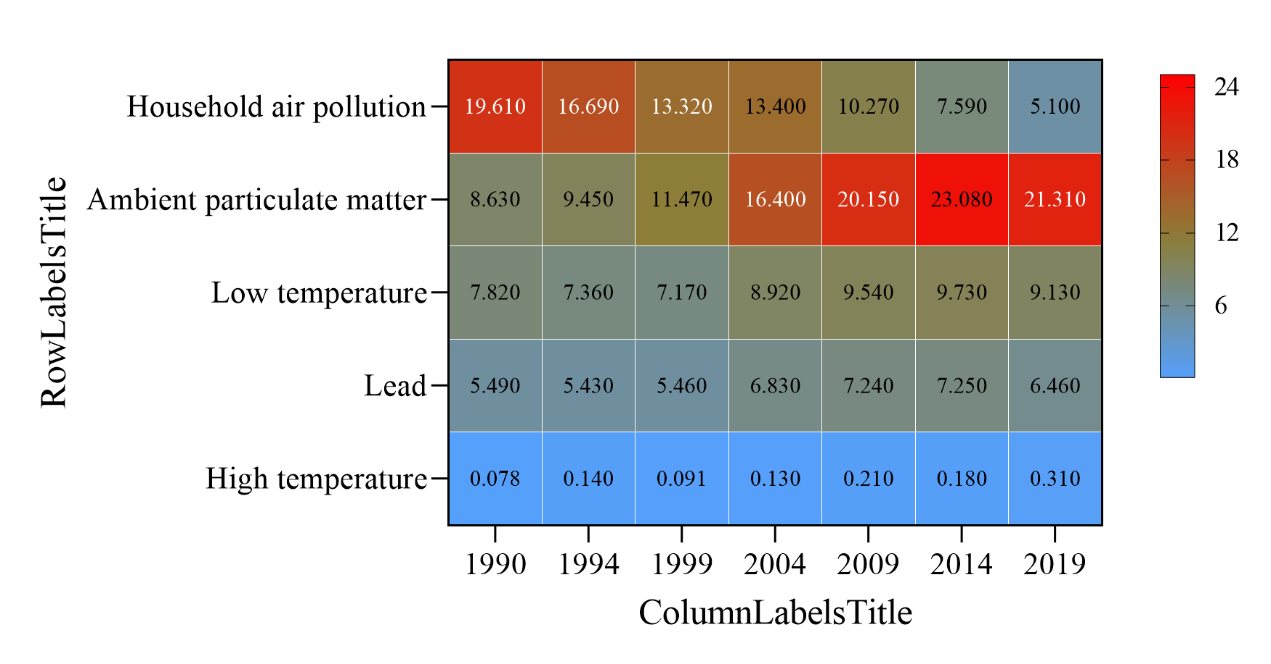


Fig. S3 The age-standardized mortality of IHD due to environmental risk factors in China in 1990, 1994, 1999, 2004, 2009, 2014, and 2019.

**Supplementary Tables**

**Table S1.** Sex-specific relative risks for age, period, and cohort effects on stroke mortality attributable to non-optimal temperature.

| **Factor** | **Mortality in males** | |  | **Mortality in females** | |
| --- | --- | --- | --- | --- | --- |
|  | **RR (95% CI)** | **P-value** |  | **RR (95% CI)** | **P-value** |
| Age |  |  |  |  |  |
| 25-29 | 0.07 (0.02-0.21) | <0.001 |  | 0.08 (0.02-0.31) | <0.001 |
| 30-34 | 0.12 (0.06-0.26) | <0.001 |  | 0.12 (0.04-0.33) | <0.001 |
| 35-39 | 0.22 (0.12-0.40) | <0.001 |  | 0.19 (0.09-0.42) | <0.001 |
| 40-44 | 0.41 (0.26-0.65) | <0.001 |  | 0.35 (0.19-0.62) | <0.001 |
| 45-49 | 0.58 (0.40-0.84) | 0.004 |  | 0.53 (0.33-0.85) | 0.008 |
| 50-54 | 0.92 (0.69-1.24) | 0.596 |  | 0.88 (0.61-1.27) | 0.494 |
| 55-59 | 1.34 (1.06-1.70) | 0.014 |  | 1.20 (0.90-1.61) | 0.216 |
| 60-64 | 1.93 (1.60-2.32) | <0.001 |  | 1.79 (1.42-2.26) | <0.001 |
| 65-69 | 2.85 (2.45-3.31) | <0.001 |  | 2.83 (2.30-3.47) | <0.001 |
| 70-74 | 4.61 (3.99-5.32) | <0.001 |  | 5.03 (4.04-6.26) | <0.001 |
| 75-79 | 6.81 (5.77-8.03) | <0.001 |  | 7.85 (6.01-10.26) | <0.001 |
| 80-84 | 10.13 (8.24-12.46) | <0.001 |  | 13.11 (9.38-18.30) | <0.001 |
| Period |  |  |  |  |  |
| 1990-1994 | 0.85 (0.72-1.01) | 0.065 |  | 1.10 (0.88-1.38) | 0.406 |
| 1995-1999 | 0.90 (0.80-1.00) | 0.056 |  | 0.99 (0.85-1.15) | 0.89 |
| 2000-2004 | 1.03 (0.96-1.11) | 0.399 |  | 1.08 (0.99-1.18) | 0.099 |
| 2005-2009 | 1.01 (0.93-1.09) | 0.820 |  | 0.98 (0.89-1.08) | 0.676 |
| 2010-2014 | 1.11 (0.99-1.25) | 0.070 |  | 0.96 (0.82-1.12) | 0.57 |
| 2015-2019 | 1.13 (0.96-1.33) | 0.151 |  | 0.91 (0.72-1.14) | 0.406 |
| Cohort |  |  |  |  |  |
| 1910-1914 | 2.99 (2.19-4.09) | <0.001 |  | 2.47 (1.57-3.89) | <0.001 |
| 1915-1919 | 2.72 (2.10-3.52) | <0.001 |  | 2.41 (1.64-3.54) | <0.001 |
| 1920-1924 | 2.40 (1.93-2.98) | <0.001 |  | 2.32 (1.66-3.24) | <0.001 |
| 1925-1929 | 2.13 (1.76-2.58) | <0.001 |  | 2.23 (1.65-3.02) | <0.001 |
| 1930-1934 | 1.83 (1.53-2.20) | <0.001 |  | 2.05 (1.52-2.75) | <0.001 |
| 1935-1939 | 1.60 (1.32-1.94) | <0.001 |  | 1.81 (1.33-2.47) | <0.001 |
| 1940-1944 | 1.34 (1.06-1.68) | 0.012 |  | 1.59 (1.11-2.27) | 0.011 |
| 1945-1949 | 1.15 (0.87-1.51) | 0.329 |  | 1.40 (0.92-2.13) | 0.113 |
| 1950-1954 | 0.97 (0.69-1.35) | 0.859 |  | 1.25 (0.76-2.04) | 0.382 |
| 1955-1959 | 0.80 (0.54-1.19) | 0.279 |  | 1.00 (0.56-1.78) | 0.991 |
| 1960-1964 | 0.66 (0.41-1.06) | 0.084 |  | 0.77 (0.39-1.53) | 0.46 |
| 1965-1969 | 0.62 (0.36-1.06) | 0.078 |  | 0.66 (0.30-1.45) | 0.306 |
| 1970-1974 | 0.51 (0.27-0.99) | 0.046 |  | 0.55 (0.21-1.44) | 0.223 |
| 1975-1979 | 0.44 (0.20-1.01) | 0.051 |  | 0.44 (0.13-1.52) | 0.192 |
| 1980-1984 | 0.44 (0.15-1.27) | 0.128 |  | 0.35 (0.06-1.99) | 0.237 |
| 1985-1989 | 0.43 (0.10-1.87) | 0.262 |  | 0.30 (0.03-3.50) | 0.336 |

**Table 3** (Continued).

| **Factor** | **Mortality in males** | |  | **Mortality in females** | |
| --- | --- | --- | --- | --- | --- |
|  | **RR (95% CI)** | **P-value** |  | **RR (95% CI)** | **P-value** |
| 1990-1994 | 0.39 (0.02-7.03) | 0.522 |  | 0.24 (0.00-30.37) | 0.566 |
| Deviance | 0.93 |  |  | 1.35 |  |
| AIC | 5.48 |  |  | 5.03 |  |
| BIC | -170.74 |  |  | -168.72 |  |

Notes: RR denotes the relative risk of stroke death attributable to non-optimal temperature in particular age, period, or birth cohort relative to the average level of all ages, periods, or birth cohorts combined.

RR, relative risk; CI, confidence interval; AIC, Akaike Information Criterion; BIC, Bayesian Information Criterion.

**Table S2.** Sex-specific relative risks for age, period, and cohort effects on IHD mortality attributable to non-optimal temperature.

| **Factor** | **Mortality in males** | |  | **Mortality in females** | |
| --- | --- | --- | --- | --- | --- |
|  | **RR (95% CI)** | **P-value** |  | **RR (95% CI)** | **P-value** |
| Age |  |  |  |  |  |
| 25-29 | 0.10 (0.03-0.34) | <0.001 |  | 0.15 (0.04-0.65) | 0.011 |
| 30-34 | 0.17 (0.07-0.41) | <0.001 |  | 0.19 (0.06-0.61) | 0.005 |
| 35-39 | 0.29 (0.15-0.57) | <0.001 |  | 0.22 (0.08-0.61) | 0.003 |
| 40-44 | 0.45 (0.26-0.79) | 0.005 |  | 0.32 (0.14-0.71) | 0.005 |
| 45-49 | 0.58 (0.36-0.93) | 0.025 |  | 0.45 (0.23-0.87) | 0.017 |
| 50-54 | 0.84 (0.57-1.23) | 0.360 |  | 0.63 (0.37-1.07) | 0.087 |
| 55-59 | 1.16 (0.85-1.59) | 0.343 |  | 0.87 (0.57-1.34) | 0.532 |
| 60-64 | 1.58 (1.23-2.04) | <0.001 |  | 1.46 (1.04-2.04) | 0.027 |
| 65-69 | 2.28 (1.86-2.80) | <0.001 |  | 2.39 (1.81-3.16) | <0.001 |
| 70-74 | 3.60 (3.01-4.31) | <0.001 |  | 4.48 (3.42-5.85) | <0.001 |
| 75-79 | 5.73 (4.74-6.92) | <0.001 |  | 7.86 (5.78-10.70) | <0.001 |
| 80-84 | 10.46 (8.33-13.13) | <0.001 |  | 15.89 (10.89-23.18) | <0.001 |
| Period |  |  |  |  |  |
| 1990-1994 | 0.77 (0.62-0.94) | 0.012 |  | 0.99 (0.74-1.32) | 0.951 |
| 1995-1999 | 0.74 (0.63-0.86) | <0.001 |  | 0.81 (0.66-1.00) | 0.047 |
| 2000-2004 | 0.87 (0.78-0.98) | 0.021 |  | 0.81 (0.70-0.94) | 0.006 |
| 2005-2009 | 1.05 (0.93-1.17) | 0.441 |  | 1.06 (0.92-1.22) | 0.411 |
| 2010-2014 | 1.35 (1.17-1.56) | <0.001 |  | 1.20 (0.99-1.46) | 0.060 |
| 2015-2019 | 1.44 (1.19-1.75) | <0.001 |  | 1.20 (0.91-1.59) | 0.196 |
| Cohort |  |  |  |  |  |
| 1910-1914 | 2.18 (1.50-3.18) | <0.001 |  | 1.80 (1.05-3.08) | 0.031 |
| 1915-1919 | 2.13 (1.57-2.89) | <0.001 |  | 1.86 (1.19-2.91) | 0.006 |
| 1920-1924 | 2.05 (1.58-2.64) | <0.001 |  | 1.86 (1.27-2.74) | 0.002 |
| 1925-1929 | 1.94 (1.55-2.42) | <0.001 |  | 1.98 (1.41-2.79) | <0.001 |
| 1930-1934 | 1.76 (1.43-2.17) | <0.001 |  | 2.00 (1.44-2.77) | <0.001 |
| 1935-1939 | 1.62 (1.31-2.00) | <0.001 |  | 1.90 (1.35-2.67) | <0.001 |
| 1940-1944 | 1.37 (1.05-1.79) | 0.019 |  | 1.73 (1.15-2.59) | 0.009 |
| 1945-1949 | 1.19 (0.86-1.66) | 0.287 |  | 1.55 (0.94-2.53) | 0.083 |
| 1950-1954 | 1.04 (0.70-1.55) | 0.847 |  | 1.42 (0.78-2.57) | 0.250 |
| 1955-1959 | 0.89 (0.56-1.42) | 0.624 |  | 1.19 (0.59-2.42) | 0.630 |
| 1960-1964 | 0.75 (0.44-1.30) | 0.312 |  | 0.93 (0.40-2.15) | 0.857 |
| 1965-1969 | 0.71 (0.38-1.32) | 0.276 |  | 0.78 (0.30-2.03) | 0.616 |
| 1970-1974 | 0.58 (0.28-1.23) | 0.157 |  | 0.66 (0.21-2.08) | 0.478 |
| 1975-1979 | 0.50 (0.20-1.22) | 0.125 |  | 0.50 (0.12-2.14) | 0.352 |
| 1980-1984 | 0.46 (0.15-1.43) | 0.180 |  | 0.34 (0.05-2.41) | 0.281 |
| 1985-1989 | 0.45 (0.10-2.05) | 0.300 |  | 0.27 (0.02-3.68) | 0.328 |
| 1990-1994 | 0.39 (0.02-8.04) | 0.544 |  | 0.21 (0.00-32.67) | 0.543 |
| Deviance | 0.49 |  |  | 1.68 |  |
| AIC | 4.87 |  |  | 4.34 |  |

**Table 3** (Continued).

| **Factor** | **Mortality in males** | |  | **Mortality in females** | |
| --- | --- | --- | --- | --- | --- |
|  | **RR (95% CI)** | **P-value** |  | **RR (95% CI)** | **P-value** |
| BIC | -170.57 |  |  | -169.39 |  |

Notes: RR denotes the relative risk of IHD death attributable to non-optimal temperature in particular age, period, or birth cohort relative to the average level of all ages, periods, or birth cohorts combined.

RR, relative risk; CI, confidence interval; AIC, Akaike Information Criterion; BIC, Bayesian Information Criterion.
